# Os hospitais em Portugal no século XIX e na primeira metade do século XX

**DOI:** 10.1590/S0104-59702024000100038

**Published:** 2024-10-04

**Authors:** Alexandra Esteves

**Affiliations:** i Professora, Departamento de História/Universidade do Minho. Braga – Portugal. alexandraesteves@ics.uminho.pt

**Keywords:** Hospitais, Portugal, Assistência, Saúde, Hospitals, Portugal, Health care, Health

## Abstract

Desde a época moderna, em Portugal, a assistência à doença foi prestada, sobretudo, pelos hospitais, muitos deles criados pelas Misericórdias. No entanto, a criação de novos hospitais no século XIX e nas primeiras décadas do século XX não trouxe, necessariamente, uma melhoria no tratamento dos doentes, visto que, salvo raras exceções, eram espaços pequenos, mal equipados e carentes de recursos humanos e materiais. O objetivo deste texto é apresentar uma reflexão sobre a resposta hospitalar em Portugal entre 1834 e as primeiras décadas do século XX. Pretendemos apontar as mudanças e continuidades que, nesse período, ocorreram no campo da assistência à doença, fazendo referência à criação de diversas instituições.

## Abstract (en)

Since the modern era, healthcare in Portugal has been provided primarily by hospitals, many of which were founded by the Sisters of Mercy and known as Misericordias. However, the creation of new hospitals in the nineteenth century and first half of the twentieth century did not necessarily yield improved patient care, since these institutions tended to be small, poorly equipped, and lacking in human and material resources. This study presents a reflection on developments in Portuguese hospitals from 1834 through to the first decades of the twentieth century, drawing on data on several institutions to identify changes and continuities in the healthcare offered.

As mudanças políticas ocorridas em Portugal ao longo dos séculos XIX e XX, bem como os avanços alcançados no campo da ciência e, particularmente, no âmbito da medicina, também se refletiram no universo hospitalar. No entanto, essas alterações aconteceram de forma paulatina, num Portugal sem Estado social e onde o poder político, materializado sob a forma de diferentes regimes (monarquia constitucional, República, ditadura militar e Estado Novo), mostrou-se mais interventivo no campo da saúde pública, o que se traduziu numa intensa produção legislativa, nem sempre condizente com o panorama nacional e nem sempre aplicada de modo a dar resposta às necessidades das populações em matéria de assistência hospitalar (Alves, Carneiro, 2013; Alves, 2018; Ribeiro 2018).^1^ Nesse domínio, a atuação do Estado português foi, de fato, tardia e inconsequente.^2^ Juntamente com outros fatores, as lacunas da intervenção do Estado na assistência à saúde devem ser consideradas para explicar a elevada mortalidade infantil, a forte incidência de doenças infeciosas, as dificuldades do combate contra a tuberculose ou o modo de encarar e tratar a doença mental (Gato, 2013).

Nascido no século XIX, fruto da ação bismarckiana que visava impedir o avanço do socialismo e das reivindicações do operariado, e numa economia industrializada que expunha o novo trabalhador – o operário – a um conjunto de riscos que se pretendia evitar, o Estado social emerge com os seguros sociais obrigatórios, entre os quais consta a proteção à saúde (Carreira, 1996; Silva, 2013). Bismarck definia a sua política como um “socialismo de Estado”, reforçando o poder central do Estado. A Alemanha adota, então, uma lei inovadora que obriga os empregadores a contribuir para um esquema de seguro-doença. De fato, os sistemas de saúde desenvolvem-se com a industrialização, e, apesar das suas limitações, o sistema bismarckiano será adotado e melhorado pelos países escandinavos e pelos Estados Unidos (Pereirinha, 2018). É de salientar que a implementação dessas medidas, sobretudo a obrigatoriedade do seguro, representa um afastamento em relação à política liberal, que defendia a responsabilização individual dos riscos (Pereirinha, 2018). Também a Inglaterra irá aplicar o sistema bismarckiano a partir de 1911, que será melhorado por William Beveridge, que estabeleceu um modelo de maior intervenção do Estado, nomeadamente por meio da promoção de políticas igualitárias.

A instabilidade política que marcou o país, sobretudo nas últimas décadas do século XIX, a que se juntou uma conjuntura internacional que desembocou na Primeira Guerra Mundial, dificultou a concretização de reformas tidas como indispensáveis. As respostas no domínio assistencial multiplicaram-se, mas não foram acompanhadas, por exemplo, pelo reforço da formação de pessoal para exercer funções nas instituições que foram sendo criadas (Alves, 2010). É de referir que, em oitocentos, a saúde pública visava, essencialmente, a educação sanitária, o saneamento, a higiene materno-infantil, a sanidade mental, a profilaxia das doenças consideradas sociais e transmissíveis, o controlo sanitário das fronteiras, a hidrologia médica e as estações balneares, bem como a fiscalização do comércio de medicamentos.

O Estado, apesar das reformas preconizadas, como a de 1901, que lhe atribuía responsabilidades em matéria de saúde pública, manteve-se, até à implantação do regime republicano, como um coordenador de iniciativas assistenciais promovidas por outras entidades, sendo poucas as da sua autoria (Ribeiro, 2018). É de referir que, com monarquia constitucional, apareceram novas instituições para responder às carências na área da saúde, juntando-se às existentes no Antigo Regime, como as Misericórdias, Confrarias e Ordens Terceiras. No entanto, o Estado tornar-se-á cada vez mais presente no campo social, particularmente na área da saúde (Lopes, 2013).

A implantação da República em 1910 trouxe consigo uma visão distinta da intervenção estatal, materializada na Constituição de 1911, onde está consagrado o direito à assistência pública, que se traduzirá no lançamento de reformas e na produção de legislação, cuja concretização irá esbarrar na instabilidade política do regime. Em 1926, entretanto, haverá nova reforma, preconizada por Ricardo Jorge, que visava conferir maior autonomia às instituições prestadoras de cuidados de saúde dos concelhos (Costa, 2009, p.75).

Até à década de 1930, grande parte da resposta na área da saúde, em termos institucionais, passava pelo hospital geral, embora se assista à criação de outros organismos especializados, sendo que os hospitais portugueses, na sua maioria geridos pelas Misericórdias, não acompanhavam os progressos já conseguidos nos congéneres europeus.

Para sustentar a nossa análise e apresentar um quadro o mais holístico possível da realidade hospitalar portuguesa no período selecionado, recorremos a um conjunto variado de fontes, nomeadamente à legislação promulgada, a debates parlamentares, a estatísticas e relatórios produzidos no âmbito da saúde, à imprensa médica e a obras de clínicos que escreveram sobre a realidade hospitalar do seu tempo, bem como a anuários estatísticos e à documentação produzida por alguns estabelecimentos hospitalares.

O hospital, como instituição, tem a sua própria história, ao longo da qual desempenhou diferentes funções, antes de ser reconhecido como a chave central de um sistema de cuidados de saúde, de desenvolvimento tecnológico e de formação de profissionais da área. Foi espaço que serviu para acolher peregrinos e marginais, bem como para socorrer quem buscava alimento e descanso (Riva, Cesana, 2013).

Desde a década de 1990, os hospitais têm sido objeto de pesquisas por parte da historiografia portuguesa, sobretudo por historiadores que se têm dedicado ao estudo das Misericórdias e da sua ação no campo da saúde, no cumprimento de uma das suas obras (“curar os enfermos”), destacando-se a época moderna como o tempo histórico desses trabalhos (Abreu, 2013; Araújo, 2022; Lopes, 2000; Sá, 2020). Nos últimos vinte anos, no entando, os estudos sobre a realidade hospitalar portuguesa da época contemporânea já são em número significativo, correspondendo à necessidade de conhecer mudanças e continuidades em relação ao período histórico anterior, bem como caracterizar uma realidade que estava praticamente esquecida (Couto, 2009; Silva, 2017; Correia, 2015, 2016; Esteves, 2016; Lopes, 2012; Fernandes, 2016; Anica, 2006; Silva, 2014b; Araújo, 2010). Importa, a propósito, relevar as obras de médicos que, nos anos 1950 e 1960, se predispuseram a contar a história dos hospitais em Portugal, embora condicionada pelo contexto do Estado Novo, sendo disso exemplo as publicações de Fernando da Silva Correia, médico e higienista português (Almeida, 2023). Esse médico denunciava, em 1938, na sua obra *Portugal sanitário,* a insuficiência de respostas na área da saúde, que, ao tempo, estavam a cargo de hospitais gerais ou especializados, sanatórios, dispensários, médicos do partido, parteiras municipais, associações mutualistas. A intervenção do Estado na assistência à saúde, tanto a nível central como local, era reduzida. Câmaras Municipais, Juntas Gerais de Distrito e Juntas de Freguesia, aliás, não desconsideravam, mas, pelo contrário, denunciavam as carências que afetavam a população no que respeita ao acesso a cuidados médicos.

Durante o Estado Novo, persistiram, *grosso modo*, as preocupações no domínio da saúde pública, mais evidentes com algumas enfermidades, de que é exemplo a tuberculose, e que levarão à criação de organismos especializados (Cerqueira, 2016). São definidos os responsáveis pelos encargos da assistência: além dos próprios assistidos, os seus familiares próximos e, no caso de impossibilidade desses, as instituições corporativas, as Câmaras Municipais e o Estado.

Observando a Figura 1, respeitante ao número de médicos em Portugal nos finais da década de 1920 e nos anos de 1931, 1934 e 1938, construída com dados colhidos por Fernando da Silva Correia em diferentes fontes, é possível constatar não só a escassez de médicos, mas também a sua concentração nas cidades onde existiam faculdades de medicina: Lisboa, Porto e Coimbra. Além disso, tratava-se de uma profissão mal remunerada, tendo em conta o seu estatuto social, a relevância da função e a exigência académica e profissional, sendo a situação particularmente grave nos meios rurais mais isolados, onde o exercício do ofício era ainda menos atrativo.

**Figura 1:**
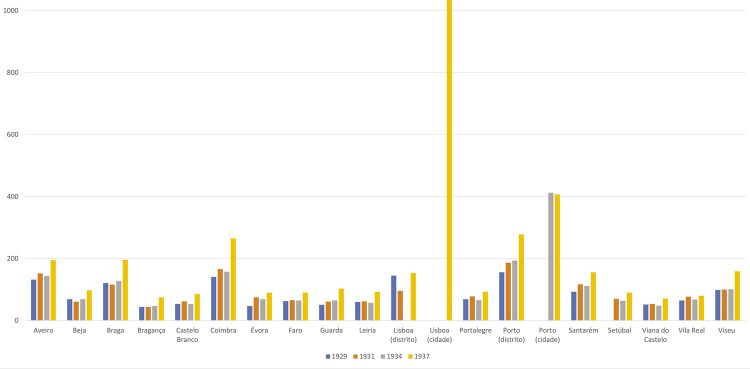
: Médicos em Portugal nos anos 1929, 1931, 1934 e 1937 (Correia, 1938)

Como é de prever, a população com menos recursos era a mais afetada pelos problemas na área da saúde, dado que os mais abastados podiam recorrer à assistência domiciliária. Aliás, até praticamente a segunda metade do século XX, quem tinha mais posses evitava o ambiente hospitalar, dada a sua associação com a pobreza e o desamparo, apesar da aposta na criação de enfermarias privadas, que, no entanto, eram pagas.

No século XX, as Misericórdias portuguesas passaram por mudanças e viveram momentos complicados sob o ponto de vista financeiro, devido, sobretudo, aos encargos inerentes à gestão dos hospitais. Acerca das Santas Casas e do seu papel no campo da saúde, Fernando da Silva Correia (1938, p.311), além de considerar que precisavam atualizar os seus serviços, afirmou que

a quase totalidade das misericórdias pouco mais têm do que o nome, ou exercem quando muito e à custa do Estado, a assistência hospitalar apenas modestamente e sem que à dedicação dos médicos e os mesários corresponda o auxílio misericordioso dos que deviam manter conforme a sua longa tradição.

Não era a única voz crítica sobre o papel das Misericórdias na administração hospitalar. Reclamava-se, por exemplo, que tivessem em atenção a opinião técnica dos médicos para a organização dos seus serviços (Costa, 2009, p.87). Tratava-se de colocar o ónus nas instituições gerentes, numa época em que prevalecia uma visão assistencialista da saúde, e o Estado não pretendia assumir responsabilidades na matéria. Aliás, as Misericórdias serão consideradas pelo Estado Novo peças centrais na promoção da assistência a nível concelhio, assumindo o Estado um papel supletivo (Sá, Lopes, 2008).

## O quadro hospitalar de Lisboa

Na primeira década do século passado, apesar da existência do que poderíamos interpretar como sendo uma ampla resposta hospitalar, composta pelos hospitais de São José, Desterro, S. Lázaro, D. Estefânia, Rainha D. Amélia, Rilhafoles e Odivelas, a cidade de Lisboa dava mostras de estar à beira da rutura no que concerne à capacidade de acolhimento e tratamento de doentes. Tornava-se urgente, por isso, a criação de mais estabelecimentos, até porque as soluções até então aventadas, que previam a instalação de hospitais provinciais, o aumento de subsídios a Câmaras Municipais e Misericórdias, bem como a assistência domiciliária ou uma mais eficaz distribuição dos doentes, não resolviam o problema do fluxo contínuo de enfermos que chegava a Lisboa (A Medicina Contemporânea, abr. 1913, p.143).

A sobrelotação dos hospitais da capital, designadamente do Hospital de São José, era atribuída, igualmente, à deficiente gestão dos ingressos, considerando-se que muitos dos doentes não deveriam ser internados e que outros permaneciam demasiado tempo nas enfermarias. É de notar que os pacientes eram admitidos no Hospital de São José e só depois eram encaminhados para outros hospitais, conforme a natureza da patologia diagnosticada. Os doentes pobres estavam isentos de pagamento do internamento, mediante a apresentação de atestado de pobreza (Cabral, 1908).

O crescente aumento da população hospitalar era acompanhado pelo aumento da exigência dos requisitos a cumprir pelos próprios hospitais, ditados pelos avanços entretanto registados no campo da medicina, o que, por sua vez, se traduzia num claro acréscimo da despesa nos respetivos orçamentos. Por outro lado, o surgimento de novas enfermidades e a ocorrência de surtos epidémicos levaram à multiplicação de legislação e regulamentos, tendo em vista remediar a situação e melhorar os serviços, bem como a intervenções em edifícios transformados em hospitais, quase todos antigos conventos.

Na época, alguns clínicos retratavam o Hospital de São José como “um viveiro de micróbios” e, numa linguagem hiperbólica, denunciavam os casos de pessoas que eram admitidas com doenças banais e que acabavam por sair em caixões. Pelo decreto de 26 de novembro de 1851, que havia reestruturado a beneficência pública, o Hospital de São José deixou de ser tutelado pela Misericórdia de Lisboa, passando para as mãos do Estado (Almeida, 2017). Note-se que esse estabelecimento contava, em 1890, com 882 camas. Além desse, existiam quatro anexos: São Lázaro, Desterro, Rilhafoles e Estefânia. Contudo, o número crescente de internamentos fazia crescer os ecos de insatisfação diante da resposta hospitalar da capital não só por parte dos clínicos, mas também da população em geral.

As críticas ao quotidiano hospitalar não se limitavam aos estabelecimentos da capital. Em 1898, considerava-se que os hospitais de Coimbra não eram consentâneos com as necessidades do ensino da medicina, o número de camas era insuficiente para fazer face à procura, e as instalações não primavam pela higiene (A Medicina Contemporânea, jul. 1898, p.194). A situação era ainda mais grave nas localidades onde não havia qualquer estrutura hospitalar, ficando as populações deixadas à sua sorte. Essa era a realidade de muitas sedes de concelho em Portugal ainda em inícios do século XX.

Os últimos anos do século XIX e as primeiras décadas do século XX foram marcados pela atuação de dois enfermeiros-mores, que foi fundamental para atenuar o impacto das lacunas existentes nos hospitais de Lisboa. São eles Ferraz de Macedo, que exerceu o cargo entre 1890 e 1900, e Curry Cabral, que desempenhou funções entre 1901 e 1910. O primeiro, apesar de não ter conseguido apoio por parte do poder público para efetuar uma série de intervenções julgadas indispensáveis, empenhou-se no melhoramento da limpeza das instalações, na criação de condições para o isolamento dos enfermos portadores de doenças infeciosas e em proporcionar mais conforto aos doentes, cuidando, por exemplo, da sua alimentação. É de relevar, ainda, a sua intervenção na instalação de hospitais provisórios, de consultas externas e no início dos serviços do Instituto Bacteriológico e do serviço antidiftérico, entre outras reformas importantes para regular o funcionamento hospitalar. Por sua vez, Curry Cabral apostou na reorganização dos serviços hospitalares, empenhou-se na fundação do Hospital do Rego e na construção do Hospital de Santa Marta, entre outras intervenções.

É de assinalar, entretanto, a reforma de 1901, estabelecida pelo decreto de 24 de dezembro de 1901, e a criação, por decreto de junho de 1902, publicado em *Diário da República* em 2 de julho do mesmo ano, de uma comissão para conservação, reparação e melhoramentos dos hospitais. A reforma material das instituições hospitalares foi preconizada por Hintze Ribeiro e previa a criação de novos estabelecimentos e a realização de melhoramentos nos existentes (Cabral, 1908). Por sua vez, a referida comissão, da qual faziam parte Curry Cabral, Luiz de Mello Correia Pereira, engenheiro civil, e José Teixeira Gomes, que era secretário da administração dos hospitais, propôs um plano de internamento com base na natureza das doenças. Assim, os utentes que indiciassem patologias que não requeriam tratamento especializado seriam encaminhados para o Hospital de São José; os doentes psiquiátricos, para Rilhafoles; e os de pediatria, para o D. Estefânia. Previa-se também a construção de dois novos hospitais, sendo um para portadores de doenças infectocontagiosas e outro para doenças venéreas e urinárias, e ainda de uma maternidade, que não existia na cidade de Lisboa.

Quando assumiu funções como enfermeiro-mor, Curry Cabral encontrou uma situação catastrófica sob o ponto de vista financeiro. A administração estava a cargo do enfermeiro-mor, de um adjunto e de um secretário, que estavam na dependência da Direção-geral da Saúde e da Assistência. A sua ação foi de inegável relevância, mas foi exonerado com a chegada do novo regime, no decurso do qual outros médicos se destacaram na tentativa de melhorar os hospitais civis de Lisboa, como foi o caso de Francisco Gentil.

No sentido de resolver os problemas que dificultavam o funcionamento das unidades hospitalares da capital, foi aprovado o decreto 1:1137, de 27 de novembro de 1914, publicado no dia 3 de dezembro desse ano, que previa a criação dos hospitais civis de Lisboa, que englobavam os que já estavam incluídos na designação, já utilizada, “Hospital de São José e anexos”, os hospitais de São José (1775), São Lázaro (1841), Desterro (1857), D. Estefânia (1877), Arroios (1892), Santa Marta (1903), Rego (depois chamado Curry Cabral, 1906) e, mais tarde, o Hospital de Santo António dos Capuchos.

Segundo o referido decreto, a administração desses estabelecimentos ficava a cargo de uma comissão composta pelos diretores dos hospitais civis e por um administrador adjunto, que desempenharia também as funções de secretário. Cada um dos hospitais teria o seu diretor, com exceção dos hospitais de São Lázaro e do Desterro, cujo diretor seria o mesmo do Hospital de São José. Cabia à referida comissão diretiva eleger, entre os seus membros, um presidente, superintender a administração técnica e financeira dos hospitais de Lisboa e elaborar regulamentos, que seriam submetidos à aprovação governamental (A Medicina Contemporânea, nov. 1914, p.404).

Motivos de ordem vária impediram o funcionamento da referida comissão, o que levará, por decreto de 1918, à criação de uma direção única “para a gerência técnica e administrativa dos hospitais civis de Lisboa, nos termos e com as faculdades que ao diretor de S. José e anexos cabiam anteriormente ao decreto de 27 de novembro de 1914” (A Medicina Contemporânea, fev. 1918, p.48). Segundo as novas disposições, publicadas no *Diário do Governo* de 12 de julho de 1918, os hospitais civis de Lisboa seriam uma entidade técnica e administrativa autónoma, dependente do Ministério do Interior e constituída pelas seguintes instituições, para além do hospital de São José: S. Lázaro, escola de enfermagem com serviço clínico; Desterro, com dermatologia, urologia, sifiligrafia e doenças venéreas; Estefânia, para mulheres e crianças; Arroios, com serviço para leprosos; Rego, para as doenças infetocontagiosas; e dispensário popular de Alcântara. Admitia-se a possibilidade de a esses institutos serem agregados outros que, entretanto, fossem criados e que não pertencessem à Faculdade de Medicina de Lisboa. Por sua vez, o Hospital Escolar de Santa Marta, assim como o Manicómio Bombarda e os serviços de hospitalização antirrábica e antidiftérica do Instituto Bacteriológico Câmara Pestana ficavam sob gestão da Faculdade de Medicina de Lisboa (A Medicina Contemporânea, jul. 1918, p.238).

Na Primeira República, além dos hospitais civis de Lisboa, existiam o Hospital Militar, destinado aos doentes da guarnição de Lisboa; o Hospital da Marinha, para oficiais e praças da Armada, que dispunha, além das enfermarias de medicina e cirurgia e de isolamento, de serviços destinados a tuberculosos e portadores de doenças de pele. Este estabelecimento também podia atender outras pessoas. Havia ainda o Hospital Colonial, o Hospital do Repouso, que era mantido pela Assistência Nacional aos Tuberculosos, os hospitais de Idanha e Telhal para doentes mentais, os hospitais da Ordem Terceira de São Francisco de Jesus e da Ordem do Carmo e o Hospital de Nossa Senhora da Saúde para crianças (A Medicina Contemporânea, jan. 1920, p.21).

A cidade contava com outras respostas no campo da assistência à saúde, nomeadamente da Misericórdia de Lisboa, que dispunha de serviços médicos internos e externos. Os primeiros eram prestados no posto permanente de socorros médicos, que dispunha, entre outras valências, de uma seção de vacinação; os serviços externos tinham lugar nos dispensários ou por meio de visitas domiciliárias. Também a Assistência Nacional aos Tuberculosos dava consultas na sua sede (Instituto Tuberculoso). Por outro lado, a Associação da Cruz Vermelha tinha dois postos de socorros, e no dispensário de Alcântara havia consultas para crianças. Por último, são de referir os sanatórios de Carcavelos, gerido pela Assistência Nacional aos Tuberculosos, e de Sant’Ana, mantido pela família Biester Chamiço (A Medicina Contemporânea, jan. 1920, p.22).

De fato, analisando a resposta hospitalar existente nas primeiras três décadas do século XX, tal como sucedia com os médicos, também no que concerne aos hospitais, Lisboa, Porto e Coimbra eram as cidades mais bem apetrechadas, o que não significava que fossem suficientes. Aliás, no primeiro Congresso da União Nacional, em 1934, foi abordada a falta de resposta hospitalar nas zonas urbanas (Almeida, 2017).

No respeitante a Lisboa, as instituições mencionadas no Quadro 1 eram dirigidas por diferentes entidades. O Hospital de Santa Marta, o Instituto Oftalmológico Dr. Gama Pinto e o Instituto Câmara Pestana eram geridos pela Faculdade de Medicina da capital; os hospitais de São José, Santo António dos Capuchos, Arroios, Desterro, Estefânia, Curry Cabral, a Maternidade Magalhães Coutinho e o Manicómio Bombarda eram administrados pela Direção-Geral dos Hospitais Civis; o Ministério da Guerra tinha a seu cargo os hospitais da Marinha e de Belém; a gestão do Hospital Colonial estava a cargo da Escola de Medicina Tropical; o Hospital da Misericórdia era gerido pela Santa Casa de Lisboa, e as restantes instituições eram particulares.

**Quadro 1:** : Hospitais existentes na cidade de Lisboa, 1938

Hospital	Número de camas
Santa Marta	470
São José	873
Santo António dos Capuchos	793
Arroios	192
Desterro	407
Estefânia	413
Curry Cabral	481
Instituto Oftalmológico Dr. Gama Pinto	120
Instituto Câmara Pestana	55
Hospital da Misericórdia	170
Maternidade Alfredo da Costa	300
Maternidade Magalhães Coutinho	142
Maternidade Bensaúde	63
Hospital da Ordem Terceira do Carmo	10
Hospital da Ordem Terceira de S. Francisco da Cidade	51
Hospital de Jesus	50
Hospital de Nossa Senhora da Saúde	40
Hospital de São Luiz	23
Hospital Inglês	12
Hospital Alemão	8
Hospital Israelita	17
Manicómio Bombarda	1.050
Hospital Militar da Estrela	595
Hospital Militar de Belém	282
Hospital da Marinha	250
Hospital Colonial	74

Fonte: Correia (1938, p.321).

## O quadro hospitalar do Porto

Na primeira metade do século XX, a situação da cidade do Porto era bastante diferente da que se verificava em Lisboa. Além da existência de menos camas, a resposta hospitalar era, em grande medida, assegurada pela Santa Casa da Misericórdia, conforme se pode ver no Quadro 2. Essa instituição tinha a seu cargo quatro hospitais, sendo um deles o Hospital de Santo António, a principal unidade de saúde da cidade. A Santa Casa portuense era ainda responsável pelos hospitais dos Convalescentes, dos Entrevados e pelo Hospital Conde de Ferreira, instituição que abriu portas em 1883, para acolher os doentes mentais do Norte do país. Além da Misericórdia, outras confrarias e ordens terceiras se destacaram na gestão de instituições hospitalares. O governo civil tinha a seu cargo o Hospital de Santa Clara; a Direção-geral de Assistência geria a Maternidade Júlio Dinis; a Direção-geral de Saúde administrava o Hospital Joaquim Urbano; e o Ministério da Guerra dirigia o Hospital Militar.

**Quadro 2:** : Hospitais existentes na cidade do Porto, 1938

Hospital	Número de camas
Santo António	922
Maria Pia	100
Ordem Terceira da Trindade	91
Santa Maria	104
Santa Clara	70
Nossa Senhora da Lapa	60
Ordem Terceira de São Francisco	40
Hospital do Terço e da Caridade	59
Ordem Terceira do Carmo	98
Maternidade Júlio Dinis	110
Joaquim Urbano	200
Casa de Saúde da Boavista	100
Hospital de Alienados Conde de Ferreira	766
Hospital dos Convalescentes	-
Hospital dos Entrevados	-
Hospital Militar Principal	532

Fonte: Correia (1938, p.322).

Recuando ao século XIX e ao papel central do Hospital de Santo António, importa referir que esse estabelecimento foi objeto de várias intervenções e reformas ao longo dessa centúria (Silva, 2014a; Couto, Esteves, 2018). Em 1896, foi elaborado um novo regulamento, que, entre outros objetivos, visava estreitar as relações entre o Hospital e a Escola Médico-Cirúrgica da cidade (A Medicina Moderna, 1896, p.273). No entanto, a cidade do Porto tinha carências similares às de Lisboa. A população estava a crescer, para o que contribuía o desenvolvimento da indústria e, consequentemente, do número de operários, o que, por sua vez, se refletia na procura de cuidados médicos. Assim, era cada vez mais premente a edificação de mais hospitais (Couto, Esteves, 2018).

Em 1911, foi nomeada uma comissão para estudar a situação hospitalar da cidade do Porto e fazer uma proposta ao governo que contemplasse a anexação “pedagógica das clínicas dos hospitais do Porto à respetiva Faculdade de Medicina” e, simultaneamente, fazer um plano geral da assistência médica da cidade. Era constituída pelos médicos Júlio de Matos, Magalhães Lemos, Sousa Júnior, Cândido de Pinho, Alberto d’Aguiar, Ramos de Magalhães, Carlos Albuquerque, Cálem Júnior e Vasco Nogueira (A Gazeta dos Hospitais, 1911, p.161). Nesse mesmo ano, os documentos solicitados foram entregues ao Ministério do Interior.

Em 1912, a situação era de tal forma grave no Hospital de Santo António, que os doentes esperavam vários dias para serem internados, não chegando muitos a tal destino, dado que a morte se antecipava. A solução, segundo os médicos portuenses, seria a construção de uma nova unidade hospitalar. Em 4 de junho de 1912, era publicado no *Diário do Governo* o projeto de lei que avançava com a criação de um hospital no Porto, com capacidade para duzentos doentes, e também destinado ao ensino dos alunos da Faculdade de Medicina, em local a escolher pela Câmara do Porto, tendo em consideração a opinião do diretor dessa Faculdade (A Medicina Contemporânea, fev. 1912, p.71). Em 1917, foi aprovado o projeto para a edificação do novo hospital.

Em 1915, entretanto, foi decidido que os doentes de fora da cidade do Porto que pretendessem ser atendidos no Hospital de Santo António teriam de cumprir um conjunto de requisitos: atestar a sua pobreza, identificar a doença de que padeciam, ainda que se tratasse de um diagnóstico incerto, e demonstrar a inexistência, na área de residência, de hospital que os pudesse atender (A Medicina Moderna, 1915, p.140). No mesmo ano, a Mesa da Santa Casa da Misericórdia do Porto solicitou apoio à Câmara Municipal da cidade e à Junta Geral de Distrito, dada a situação verdadeiramente aflitiva com que se confrontava, num contexto marcado pelos efeitos da Primeira Guerra Mundial, que se traduzia na carência de bens alimentares, de medicamentos e de combustível. A justificação desse pedido incluía, entre outros argumentos, o importante papel que o Hospital de Santo António desempenhava no Norte do país (A Medicina Moderna, 1915, p.290). No sentido de mitigar ou resolver as dificuldades que prejudicavam o seu funcionamento, foi decidido não receber mais de trinta doentes pobres. Os de “aquém Mondego até Vizeu” não seriam admitidos para internamento, com exceção dos que, por legado, tivessem direito a tratamento gratuito ou desejassem entrar como pensionistas de terceira classe. No caso de serem provenientes da cidade do Porto, ultrapassado o número estabelecido, só seriam aceites doentes cujo estado de saúde fosse considerado muito grave (A Medicina Moderna, 1920, p.269).

O Hospital D. Maria Pia resultou da iniciativa conjunta de um grupo de beneméritos, que decidiu fundar um hospital para tratar crianças pobres. A autorização para a sua criação foi obtida a 15 de março de 1882 e, com o patrocínio da rainha D. Maria Pia, entrou em funcionamento no ano seguinte, mas num edifício arrendado, que não estava preparado para a função. Por conseguinte, tornou-se imperioso encontrar um local apropriado para instalar essa instituição. Em 1894, num terreno doado por uma benfeitora, começou a ser erigido um hospital de raiz, que entrou em funcionamento em 1911. Na altura da Primeira Guerra Mundial, as suas instalações foram temporariamente cedidas à Delegação da Cruz Vermelha, mas continuou a receber crianças. Tratou-se de um período difícil para esse hospital, tendo sido ponderada a sua entrega à Santa Casa portuense (Pequena história..., 1882-1967). No período compreendido entre 1918 e 1924, recebeu doentes de tifo exantemático e de gripe pneumónica, epidemias que fustigaram a cidade do Porto a partir de 1918. O edifício, entretanto, foi submetido a uma série de intervenções e melhoramentos, abrindo portas novamente em 1925, agora com o apoio das Missionárias de Maria.

Em 1926, foi criado o Hospital Sanatório Semide. Tratava-se de mais uma estrutura gerida pela Santa Casa da cidade do Porto, cuja fundação se deve em grande medida à filantropia de Manuel Pinto Azevedo (A Medicina Contemporânea, nov. 1926, p.368).

Se a situação era problemática nas cidades de Lisboa e Porto, nas restantes regiões do país não era melhor. Em 1915, 63% dos doentes eram tratados em estabelecimentos de assistência privada, aos quais pertenciam 70% das camas. Dos 251 hospitais então existentes, 241 eram privados, ou seja, 96% das instituições. Refira-se ainda que 24,5% da população portuguesa vivia em concelhos que não tinham hospital (A Medicina Contemporânea, jan. 1919, p.32).

## O quadro hospitalar nacional

Ainda no início do século XX, praticamente todos os hospitais gerais eram geridos pelas Santas Casas, sendo as exceções os hospitais do Cartaxo, de Mafra, de Vila Nova de Ourém e das Caldas da Rainha (Correia, 1938). A intervenção do Estado era limitada, ficando-se pela proteção das instituições de assistência e pela fiscalização “das suas contas”, embora lhes concedessem alguns apoios (Cabral, 1908, p.629). Apenas 53 estabelecimentos tinham mais de cinquenta camas. As carências eram muitas, designadamente, referentes a instalações, que precisavam de obras urgentes, mas difíceis de realizar dadas as dificuldades económicas das Misericórdias, e a equipamentos e recursos humanos.

**Figura 2:**
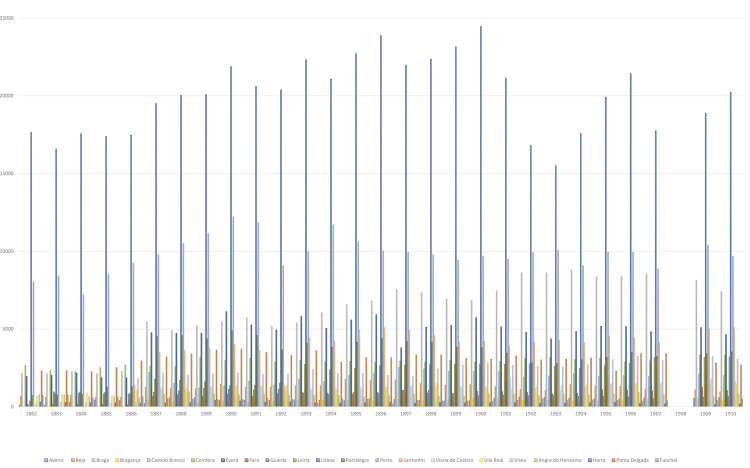
: Registo de entrada dos doentes nos hospitais no nível distrital, 1882-1910 (Anuários Estatísticos, 1882-1910)

Conforme se pode observar na Figura 2, respeitante ao período compreendido entre 1882 e 1910, todos os distritos dispunham de hospitais, mas o mesmo não acontecia, ainda nos finais do século XIX, em alguns concelhos. Essa lacuna será resolvida, ainda no final de oitocentos ou no início do século XX, graças a iniciativas filantrópicas, nomeadamente de “brasileiros de torna-viagem”. São disso exemplo os hospitais de Paredes de Coura e de Póvoa de Lanhoso, dois concelhos do Norte do país. Em matéria de assistência hospitalar, a realidade da generalidade do país era claramente deficitária quando comparada com a de Lisboa e Porto, embora existissem outros hospitais bastante concorridos, como os de Braga e de Évora.

Em 1916, o médico Reinaldo dos Santos, para resolver o problema da sobrelotação dos hospitais, nomeadamente em Lisboa e no Porto, defendia que os doentes, desde que tivessem meios, pagassem o seu internamento, e, simultaneamente, se procurasse construir hospitais em todo o país. Considerava, ainda, ser necessário reduzir o número e a duração dos internamentos, que, ao tempo, rondava os 41 dias. A consecução desse objetivo requeria, por exemplo, a regularização do funcionamento dos serviços de diagnóstico e terapêutica, a melhoria e a maximização das consultas externas (O Portugal Médico..., 1916).

Outros problemas afetavam o quotidiano hospitalar: as instalações eram desconfortáveis; faltavam balneários; havia graves dificuldades de abastecimento de água; escasseavam meios de diagnóstico, como, por exemplo, aparelhos de radiografia… (Esteves, 2013, 2015). Na mesma altura, além da insuficiência de médicos, também era notória a falta de pessoal de enfermagem, pelo que urgia a criação de novas escolas para suprir essa lacuna. Não faltavam, aliás, críticas dirigidas particularmente à enfermagem religiosa, alvo da censura de médicos, como Miguel Bombarda, que denunciavam a sua falta de formação (Silva, 2014).

A situação dos hospitais não melhorou na década de 1920, refletindo, afinal, os problemas que enfrentavam as Misericórdias portuguesas, gestoras da maior parte dos estabelecimentos do país. A crise financeira que se vivia em Portugal em 1926 afetou as instituições de assistência, designadamente as Santas Casas e os hospitais. Em 12 de janeiro de 1926, o deputado Alberto Vidal expunha, na Câmara dos Deputados, as dificuldades financeiras da Misericórdia de Ílhavo, que, apesar de ter angariado recursos para construir um hospital, que iria servir mais de vinte mil habitantes, não dispunha de meios para garantir a sua manutenção (Diário da Câmara..., 12 jan. 1926).

Na década de 1930, havia em Portugal 305 hospitais. A maioria tinha menos de cinquenta camas e apenas um médico, funcionando em condições muito precárias. As falhas de caráter estrutural, a que se juntava a escassez de recursos humanos, ganhavam maior acuidade em tempo de crise, nomeadamente quando ocorriam epidemias. As dificuldades com as instalações não afetavam apenas as pequenas instituições, sendo que, nesses casos, tinham a ver sobretudo com a sua exiguidade, mas igualmente os grandes hospitais, que eram confrontados com o problema da sobrelotação. O caso mais paradigmático era o Hospital de São José, que, ainda em finais do século XIX, era chamado a lidar com o aparecimento de doenças infeciosas na capital, o que obrigava a colocar doentes em barracas na cerca desse estabelecimento. Era a forma encontrada para isolar os infetados e evitar o convívio com a restante população hospitalar. Nem sempre, todavia, os preceitos do isolamento eram cumpridos, até pelo pessoal hospitalar (A Medicina Contemporânea, jan. 1888). Assim, não eram raros os casos de epidemias que chegavam ao interior dos hospitais e que eram responsáveis pela morte de doentes internados. A esse propósito, o médico Silva Carvalho, depois de ser declarada uma epidemia de febre puerperal no Hospital de São José, escrevia o seguinte, em 1888, nas páginas da revista *A Medicina Contemporânea*:

São múltiplas as causas do estado vergonhoso em que se encontram os nossos hospitais e de que esta epidemia é uma prova eloquente, quando se enumeram porém quase sempre esquece uma de eu nos havemos de ocupar quando tivermos tempo, porque é assumpto para demoras, é a incúria dos clínicos (A Medicina Contemporânea, jan. 1888, p.118).

Ainda na mesma década, mais precisamente em 1933, o Estado Novo, pelo decreto-lei 22.917, de 31 de julho, autorizava a construção de dois hospitais escolares, um em Lisboa e outro no Porto, que funcionariam em ligação com as Faculdades de Medicina. Contudo, foi necessário esperar praticamente 20 anos para que fosse erigido o primeiro deles: o Hospital de Santa Maria.

No decreto-lei 827 de 1937, assumia-se que a assistência privada estava mais desenvolvida que a pública, considerando-se, ainda, que a ação preventiva no campo da saúde deveria ser a grande aposta, sem, no entanto, desconsiderar a vertente curativa, então dominante. As iniciativas de índole sanitária eram separadas das atividades assistenciais, que englobavam o apoio à doença, à maternidade, à infância e à família. Segundo Gonçalves Ferreira, é a partir daqui que o Estado português arrancará com a política de construção dos grandes hospitais centrais, de criação de maternidades e de apoio às Misericórdias, às quais o Estado concedia verbas para subsidiar a prestação de cuidados de saúde (Ferreira, 2015, p.476).

Os anos 1940 serão marcados pela construção de mais estruturas hospitalares. No contexto subsequente à Segunda Guerra Mundial, a saúde assume maior protagonismo, também por parte dos Estados, e, naturalmente, o hospital ganha importância acrescida, sendo visto como um elemento central nos sistemas de saúde vigentes, particularmente nos Estados-providência. Cabia ao Estado garantir o bem-estar físico e mental dos seus cidadãos por meio da prestação de um conjunto de serviços que materializassem o conceito de saúde definido pela Organização Mundial de Saúde (OMS). Assim, o hospital será encarado como um espaço de aplicação de saberes técnicos diferenciados, e não apenas como um espaço de assistência a pobres e desvalidos que, além de outros cuidados, procuravam repouso e alimentação melhorada (Costa, 2009). Essa mudança do paradigma hospitalar acarretava mais responsabilidades e maior exigência financeira, que seria assumida pelos Estados-providência.

Entretanto, depois de ter sido discutida na Assembleia Nacional, foi publicada, em 1946, a lei 2.011, que previa uma assistência hospitalar descentralizada, como refere Andreia Almeida (2017, p.400). Segundo esse documento, o país seria dividido em zonas, regiões e sub-regiões. As primeiras, que seriam três, ficariam dotadas de hospitais centrais: o da zona Norte ficaria sediado no Porto, o da zona Centro em Coimbra, e o da zona Sul em Lisboa. Os hospitais regionais estariam nas capitais de distrito, e os sub-regionais nas sedes dos concelhos. Os serviços prestados seriam diferenciados, e sempre que o doente não encontrasse resposta no hospital sub-regional ou regional poderia ser encaminhado para o hospital central.

No que concerne ao financiamento, o Estado assumia os encargos dos estabelecimentos que eram da sua competência. Nos restantes, as responsabilidades eram assumidas em regime de cooperação (Almeida, 2017, p.403). Entretanto, pela lei 2.011, foi criada a Comissão de Construções Hospitalares. A partir daqui, os hospitais integram uma política de propaganda do Estado, que surge como impulsionador da renovação de hospitais e da criação de novas instituições. Todavia, o processo não se materializará com a rapidez desejável. Refira-se que, apesar de vários hospitais sub-regionais terem sido construídos com financiamento do Estado, a sua administração foi, na maioria dos casos, entregue às Misericórdias (Costa, 2009, p.87).

## Os hospitais especializados

A realidade dos hospitais gerais também se verificava nos hospitais especializados, nomeadamente nos sanatórios e nos manicómios, que, além de escassos, estavam concentrados nas grandes cidades. Desde o século XIX, a tuberculose constituía um flagelo de graves proporções não apenas sob o ponto de vista médico, mas também social. A Associação Nacional de Tuberculosos era a responsável pela resposta institucional à enfermidade, tanto pela criação dos dispensários antituberculose como pelo estabelecimento e a gestão de sanatórios marítimos, de altitude e de planície. A Associação era, assim, responsável, no século XX, pela gestão das seguintes instituições: Sanatório Sousa Martins (Guarda), Sanatório do Lumiar e da Ajuda (Lisboa), Sanatório Rodrigues Gusmão (Portalegre), Sanatório Dr. José de Almeida (Carcavelos), Sanatório do Outão (Setúbal) e Sanatório da Gelfa (Vila Praia de Âncora). Havia, ainda, sanatórios destinados, especificamente, a ferroviários: o Sanatório General Carmona (Paredes de Coura), o Sanatório das Penhas da Saúde (Covilhã) e o Sanatório Vasconcelos Porto (São Brás de Alportel). À Junta de Província da Beira Litoral pertenciam os sanatórios de Celas e Covões, em Coimbra. As Misericórdias de Lisboa e Porto tinham a seu cargo, respetivamente, o Sanatório de Santana e o Sanatório Rodrigues Semide. Os sanatórios do Caramulo, Marítimo do Norte e Heliantia eram de gestão particular. O número de sanatórios existentes no país era manifestamente insuficiente diante da elevada incidência da tuberculose.

No que respeita às maternidades, se não considerarmos as enfermarias de partos existentes nos hospitais de maior dimensão, podemos afirmar que são uma criação do século XX. Em 1900, Lisboa, ao contrário do que sucedia na maioria das cidades europeias, tinha apenas a enfermaria de Santa Bárbara, no Hospital de São José, para receber as parturientes.^3^ A primeira maternidade que cumpriu os requisitos para ter essa designação foi a Maternidade Magalhães Coutinho, que abriu portas em 1927. Nesse ano, surgiu a Maternidade Bensaúde, mais orientada para o recebimento de mulheres grávidas desamparadas. Já na década de 1930, entrou em funcionamento a Maternidade Alfredo da Costa. Também nessa década, surgiu, na cidade do Porto, a Maternidade Júlio Dinis.

Ao longo da década de 1930, de fato, o país foi sendo dotado de pequenas maternidades, e, no fim da primeira metade do século XX, havia mais de cem em Portugal e nas colónias, contrastando, assim, com o quadro do início dessa centúria, quando essa resposta praticamente não existia. Essa mudança traduz a implementação da medicalização do parto e a aposta no combate à elevada mortalidade infantil, que era um dos flagelos que o país enfrentava. O parto em ambiente caseiro e assistido por “parteiras curiosas” era muitas vezes apontado como uma das causas da morte de mulheres e de crianças à nascença. Os médicos insistiam, aliás, na culpabilização das “curiosas” por esses óbitos. Apesar da fundação de maternidades, se bem que em número insuficiente, muitas mulheres continuavam a preferir ter os seus filhos em casa, sendo aquelas instituições procuradas, essencialmente, por mulheres pobres e desamparadas, sem enquadramento familiar.

No início do século, os hospitais infantis também não abundavam, sendo de referir apenas o Hospital de Santo António e o Hospital de Nossa Senhora da Saúde, em Lisboa. Essa cidade enfrentava uma clara carência de respostas a parturientes, recém-nascidos e crianças, o que levou a Mesa da Misericórdia a projetar a organização de um serviço de assistência domiciliária às parturientes (A Medicina Contemporânea, set. 1907, p.294).

No atinente aos hospitais psiquiátricos, o século XX manteve as instituições do século passado, destacando-se, nesse quadro, o Hospital de Rilhafoles/Miguel Bombarda, na cidade de Lisboa, e o Hospital Conde de Ferreira, na cidade do Porto, muito marcados pela sobrelotação, particularmente o primeiro, aos quais se vão juntar o Hospital Júlio de Matos, o Hospital Sobral Cid e o Manicómio Sena. Entretanto, outras respostas foram surgindo nessa área.^4^

Ainda no século XIX, o Hospital de Rilhafoles sofreu várias intervenções, que, no entanto, não foram suficientes para resolver graves problemas que afetavam o seu funcionamento. Miguel Bombarda, que assumiu a direção da instituição em 1892, procurou introduzir algumas melhorias, tanto em nível terapêutico como organizacional. Por exemplo, em 1898, apontava o abastecimento de água ao hospital como sendo uma das carências que urgiam resolver, quando a hidroterapia era um dos tratamentos a aplicar aos alienados (A Medicina Contemporânea, jul. 1898, p.243). No entanto, o problema mais complicado que a instituição teve que enfrentar até ao fim daquele século foi o da superlotação, quando atingiu mais de setecentos doentes. As causas dessa situação estavam identificadas e não resultavam unicamente da falta de respostas, mas também do crescente internamento de indivíduos que a Justiça tinha considerado inimputáveis. Além disso, muitos dos enfermos eram de fora da cidade de Lisboa (A Medicina Contemporânea, ago. 1899, p.271).

Os estudos existentes sobre o Hospital de Rilhafoles aludem à permanência de diversas limitações nos inícios do século XX, apesar das medidas tomadas por Miguel Bombarda (Necho, 2019), como mostra o relatório referente a 1903 e 1904, apresentado por esse médico. Dos 354 doentes admitidos, 67,2% eram de fora da capital (A Medicina Contemporânea, mar. 1905, p.93). No mesmo documento, ressalta o elevado número de pensionistas, e fica claro, mediante a indicação da ocupação dos pacientes, a sua pertença a grupos sociais mais necessitados, predominando jornaleiros e trabalhadores manuais (A Medicina Contemporânea, mar. 1905, p.93). No entanto, a maioria dos indivíduos não tinha ocupação conhecida (p.93). Por outro lado, sobressai o elevado número de doentes que faleceram do que se chamava de “doenças estranhas à alienação mental” (p.93). Trata-se de uma tendência verificável nos anos seguintes, patente nos relatórios que fazem o retrato de um hospital que, para muitos, se transformava num lugar de morte, em consequência da falta de condições sanitárias (p.366).

Os problemas não se limitavam a sanatórios e hospitais psiquiátricos. Em 1900, o Hospital da Marinha apresentava carências muito semelhantes às dos seus congéneres: necessidade de obras e de ampliação das instalações para fazer face ao aumento da procura, de melhoria das condições de acolhimento e de internamento para os doentes, bem como de alargamento do pessoal hospitalar (A Medicina Contemporânea, abr. 1900, p.120).

No início do século passado, havia ainda diversas instituições privadas, que estavam concentradas nas duas maiores cidades do país. Em Lisboa, destacavam-se os hospitais de Nossa Senhora da Saúde e de Santo António, vocacionados para crianças; o Hospital do Clero; o Hospital da Ordem Terceira de São Francisco; o Hospital de Nossa Senhora da Victoria; o Hospital S. Luiz dos Franceses; o Hospital St. Louis. No Porto, havia apenas dois: o Hospital D. Maria Pia e o Hospital da Ordem Terceira da Trindade (p.331). Havia, ainda, as casas de saúde do Telhal e de Idanha, geridas pela Ordem de São João de Deus.

Sobre a assistência hospitalar nas colónias, ficamo-nos por algumas breves notas, já que a sua análise requer outra abordagem e enquadramento específico, que não cabem no presente trabalho.

Ainda afastado da Organização das Nações Unidas, na qual foi admitido em dezembro de 1955, Portugal defendeu a sua presença na OMS, que aconteceu a partir de 1948. O país estava representado neste organismo pelo diretor-geral de Saúde, Augusto da Silva Travassos, por Francisco Cambournac, António Carvalho Dias e Bernardino Álvaro Vicente de Pinho. Uma das primeiras preocupações dessa delegação consistiu em demonstrar que as colónias portuguesas não eram mais que províncias cujos habitantes tinham direitos iguais aos dos residentes na metrópole.

Em 1949, Henrique Galvão, na condição de deputado à Assembleia Nacional, denunciava a situação dos hospitais de Angola, onde, no seu entender, tudo faltava. Como seria expectável, tal denúncia foi refutada pelo regime vigente (Diário das Sessões, 1 abr. 1949, p.427). Em 1952, existiam, no conjunto das colónias portuguesas, 107 hospitais, 145 enfermarias e 603 postos sanitários. Além dessas estruturas, havia dispensários e lactários (Boletim do Instituto..., 1952, p.92). Contudo, tal como na metrópole, o número não corrige a realidade e as suas evidentes limitações.

## Considerações finais

Procurou-se, no âmbito desta reflexão, apresentar um quadro geral da realidade hospitalar portuguesa entre a segunda metade do século XIX e as primeiras décadas do século XX. Trata-se de um período marcado por mutações no campo político, mas de reduzido impacto na assistência à saúde. Se, por um lado, a instabilidade política não era favorável à realização de reformas hospitalares, sobretudo até os finais dos anos 1920, o mesmo não pode ser afirmado a partir da década seguinte, com a instalação de um regime autoritário que se irá manter por mais de quatro décadas. De fato, até finais dos anos 1920, os serviços públicos na área da saúde eram muito limitados. Para além dos hospitais civis de Lisboa, do Hospital Joaquim Urbano, na cidade do Porto, e dos hospitais escolares de Lisboa e Coimbra, o país contava com dispensários de diferentes tipologias, médicos do partido ou municipais e delegações de saúde. Com o Estado Novo, o quadro hospitalar resultará, essencialmente, da forma como era perspetivado pelo próprio regime e pelo modo como era encarada a assistência à saúde. Nesse contexto, as Misericórdias, instituições que, desde há séculos, desempenhavam um papel de revelo na prestação de cuidados hospitalares, mantinham-se como administradoras de hospitais. Apesar do crescente envolvimento do Estado na criação de novos estabelecimentos e de mudanças que acontecem, sobretudo, a partir dos anos 1960, persistia uma visão supletiva da intervenção estatal na área da saúde, como mostram estudos apresentados, nomeadamente por Ana Paula Gato.

Era a comunidade médica que, de forma mais evidente, tinha consciência das inúmeras carências que afetavam a assistência hospitalar, que impossibilitavam o acompanhamento dos progressos que iam sendo alcançados e das consequentes exigências da ciência médica. Assim, na prática, os problemas permaneciam e materializavam-se em quotidianos hospitalares a decorrer em edifícios desadequados, na escassez de profissionais de saúde e, consequentemente, na falta de uma resposta eficaz às necessidades da população. Essa realidade ajuda a perceber o quadro negro do país, ainda nos anos 1940, quando comparado com outros países europeus, no que respeita à prestação de cuidados de saúde, bem patente nos baixos indicadores que então registava, designadamente no referente à mortalidade infantil e à oferta de cuidados de saúde à generalidade da população. De fato, apesar da criação das Casas do Povo, das Casas dos Pescadores e das Caixas de Previdência, os serviços continuavam dispersos e desarticulados, com inúmeras carências e desigualdades, incapazes de chegar a toda a população, principalmente à mais necessitada.

No contexto internacional, sobretudo após Segunda Guerra Mundial, verifica-se a complexificação do hospital e também a sua afirmação como um espaço de inovação e ensino, que assume diferentes valências e que se transforma numa instituição representativa da intervenção do Estado no domínio da saúde. Esse não será o caso de Portugal até, praticamente, a criação do Serviço Nacional de Saúde, depois da queda do Estado Novo, em 1974.

Escasseiam trabalhos sobre os hospitais portugueses no século XX, bem como sobre outras respostas no campo da saúde. São necessários, portanto, estudos de caso, nomeadamente sobre os grandes hospitais entretanto edificados, como o Hospital de São João, na cidade do Porto, ou o Hospital de Santa Maria, em Lisboa, para compreender as dinâmicas dos seus primeiros anos de funcionamento e o papel efetivo do Estado português na promoção da assistência hospitalar no país, à semelhança dos que já existem para outras estruturas hospitalares construídas em Portugal no século XX, como, por exemplo, o Hospital Rovisco Pais (Xavier, 2013). A visão panorâmica auxilia no estabelecimento de uma leitura de largo espectro sobre os hospitais portugueses, mas, de fato, são precisos mais estudos de caso, como os que já existem para outras geografias europeias e para outras cronologias da realidade portuguesa, que permitam chegar a outras conclusões sobre o quadro hospitalar de Portugal, nomeadamente sobre a desigualdade no acesso aos cuidados de saúde.

## NOTAS

1 A saúde pública conheceu importantes avanços no século XIX, tendo como palco principal a Inglaterra. Entretanto, outros Estados a seguiram nas preocupações sanitárias (Stearns, Koella, 2010). Os Estados Unidos, a Alemanha e a França foram alguns deles.

2 Apesar de algumas melhorias que se registaram com o fim da Segunda Guerra Mundial, os indicadores sanitários arrastavam Portugal para um panorama idêntico ao dos demais países europeus nos finais do século XIX (Viegas, Frada, Miguel, 2006).

3 Sobre essa temática em Portugal, leia-se Carneiro (2008).

4 O caso mais grave era o do Hospital de Rilhafoles, primeiro hospital para alienados em Portugal, que funcionava num antigo convento e que, desde a sua génese, enfrentava problemas de sobrelotação. Ainda em finais do século XIX, mais precisamente em 1886, reconhecia-se que o hospital não preenchia os critérios mínimos para o cumprimento da finalidade para a qual fora criado. Por conseguinte, foi criada uma comissão para acompanhamento de obras e para a sua avaliação (A Medicina Contemporânea, maio 1886).
